# Prognostic impact of noninvasive areas in resected pathological stage IA lung adenocarcinoma

**DOI:** 10.1111/1759-7714.14910

**Published:** 2023-04-27

**Authors:** Fumihiko Kinoshita, Mototsugu Shimokawa, Tomoyoshi Takenaka, Tatsuro Okamoto, Kenichi Taguchi, Yoshinao Oda, Tomoharu Yoshizumi

**Affiliations:** ^1^ Department of Thoracic Oncology National Hospital Organization Kyushu Cancer Center Fukuoka Japan; ^2^ Department of Biostatistics, Graduate School of Medicine Yamaguchi University Fukuoka Japan; ^3^ Department of Surgery and Science, Graduate School of Medical Sciences Kyushu University Hospital Fukuoka Japan; ^4^ Department of Cancer Pathology Laboratory National Hospital Organization Kyushu Cancer Center Fukuoka Japan; ^5^ Department of Anatomic Pathology, Graduate School of Medical Sciences Kyushu University Hospital Fukuoka Japan

**Keywords:** lung adenocarcinoma, noninvasive areas, prognostic factor, surgery, TNM classification

## Abstract

**Main Problems:**

In non‐small‐cell lung cancer, ground‐glass opacity on computed tomography imaging reflects pathological noninvasiveness and is a favorable prognostic factor. However, the significance of pathological noninvasive areas (NIAs) has not been fully revealed. In this study, we aimed to elucidate the prognostic impact of NIAs on lung adenocarcinoma.

**Methods:**

We analyzed 402 patients with pathological stage (p‐Stage) IA lung adenocarcinoma who underwent surgery in 2013–2016 at two institutions and examined the association of the presence of NIAs with clinicopathological factors and prognosis. Furthermore, after using propensity‐score matching to adjust for clinicopathological factors, such as age, sex, smoking history, pathological invasive area size, pathological T factor (p‐T), p‐Stage, and histological subtype (lepidic predominant adenocarcinoma [LPA] or non‐LPA), the prognostic impact of NIAs was evaluated.

**Results:**

Patients were divided into NIA‐present (*N* = 231) and NIA‐absent (*N* = 171) groups. Multivariable analysis showed that NIA‐present was strongly associated with earlier p‐T, earlier p‐Stage, LPA, and *epidermal growth factor receptor* mutation. Kaplan–Meier survival analysis showed that the NIA‐present group displayed a better prognosis than the NIA‐absent group in disease‐free survival (DFS) and overall survival (OS) (5‐year DFS 94.6% vs. 87.2%, 5‐year OS 97.2% vs. 91.1%). However, after adjusting for clinicopathological factors by propensity score matching, no significant differences in prognosis were identified between the NIA‐present and NIA‐absent groups (5‐year DFS 92.4% vs 89.6%, 5‐year OS 95.6% vs 94.3%).

**Conclusions:**

Our current study suggests that the prognostic impact of the presence of NIAs on lung adenocarcinoma is due to differences in clinicopathological factors.

## Graphical abstract

We analyzed 402 patients with pathological stage IA lung adenocarcinoma who underwent surgery and examined the association of the presence of pathological noninvasive areas (NIAs) with prognosis. The presence of NIAs was associated with a good prognosis; however, the prognostic impact of NIAs disappeared after adjusting for the bias of clinicopathological factors by propensity‐score matched analysis, therefore the prognostic impact of NIAs might be due to differences in clinicopathological factors.

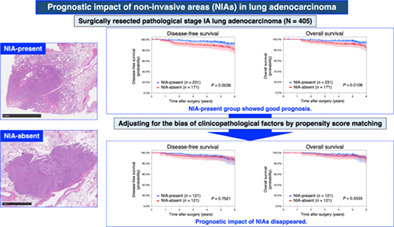

## INTRODUCTION

The tumor‐node‐metastasis (TNM) classification of lung cancer was revised to the 8th edition in 2017.^1^, ^2^, ^3^ One of the major changes in the 8th edition is that the T factor is assessed by the solid component size/invasive area size instead of the whole tumor size.^1^, ^2^, ^3^ These changes are based on the findings of several studies showing that, especially on preoperative computed tomography (CT) findings, the solid component size reflects poor prognosis and pathological malignancy more accurately than the whole tumor size.^4^, ^5^, ^6^, ^7^

In non‐small‐cell lung cancer (NSCLC), the presence of ground‐glass opacity (GGO) on CT findings has been reported to be a favorable prognostic factor for postoperative prognosis.^8^, ^9^, ^10^, ^11^, ^12^, ^13^, ^14^ However, few reports have analyzed the association between the presence of pathological noninvasive areas (NIAs) and the postoperative prognosis of NSCLC. Because the presence of GGO is a favorable prognostic factor, the presence of NIAs might also be a positive factor. However, it is highly questionable whether NIAs, which do not appear to have metastatic or invasive potential, can influence the prognosis of NSCLC. In this study, to elucidate the prognostic impact of NIAs, we analyzed how their presence contributed to the prognosis of surgically resected lung adenocarcinoma using propensity‐score matching.

## PATIENTS AND METHODS

### Study patients

This retrospective research was reviewed and approved by our institutional review boards (Kyushu Cancer Center, IRB No. 2019–56; Kyushu University Hospital, IRB No. 2019–232). A total of 462 patients with pathological stage (p‐Stage) IA NSCLC who underwent surgical resection between January 2013 and December 2016 at the Department of Thoracic Oncology, National Hospital Organization Kyushu Cancer Center and the Department of Surgery and Science, Graduate School of Medical Sciences, Kyushu University Hospital were enrolled. Of these, 409 patients were diagnosed as p‐Stage IA lung adenocarcinoma. Seven patients who received preoperative treatments were excluded and 402 patients with p‐Stage IA lung adenocarcinoma were analyzed in this research. As the routine postoperative follow‐up, physical examination, blood tests, and chest radiographs were performed at 3‐month intervals for the first 3 years followed by 6‐month intervals, and CT scans were performed at 6‐month intervals for the first 3 years followed by 1‐year intervals. Patients who met the following criteria received uracil‐tegafur as adjuvant chemotherapy: (i) pathological whole tumor size ≥2 cm, (ii) age < 76 years, (iii) Eastern Cooperative Oncology Group performance status of 0 or 1, and (iv) provided written informed consent.

We retrospectively reviewed the clinical records to assess clinicopathological factors, disease‐free survival (DFS), and overall survival (OS). Clinicopathological factors included age, sex, smoking history, radiological whole tumor size, radiological solid component size, consolidation/tumor (C/T) ratio, GGO presence, surgical procedure, pathological whole tumor size, pathological invasive area size, pathological T (p‐T) factor, p‐Stage, histological predominant subtype (lepidic, acinar/papillary, solid/micropapillary, and others), lymphatic invasion (ly), vascular invasion (v), and epidermal growth factor receptor (EGFR) mutation. The histological predominant subtypes were divided into lepidic predominant adenocarcinoma (LPA) and nonlepidic predominant adenocarcinoma (non‐LPA) groups in the analysis.

### Pathological diagnosis

**FIGURE 1:**
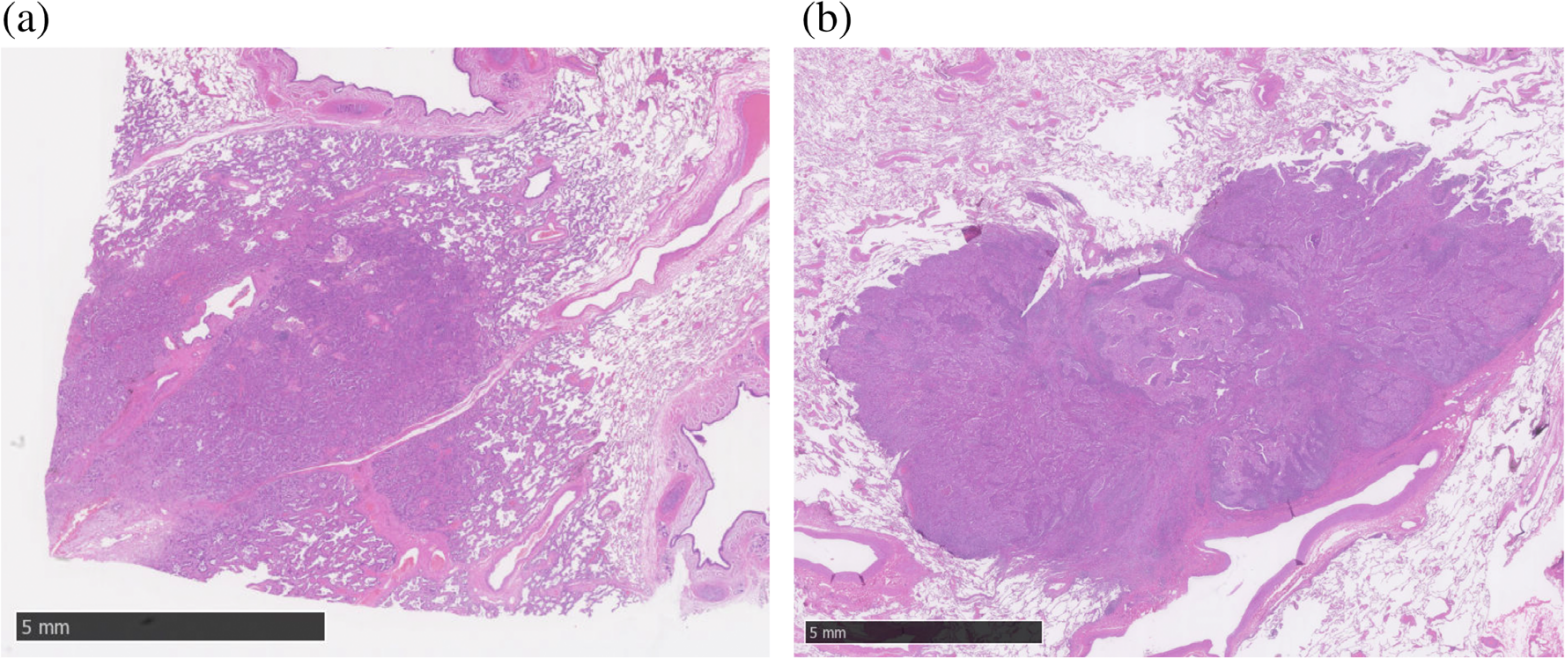
Representative histological images of NIA‐present (a) and NIA‐absent (b) tumors. NIA, noninvasive area

The pathological diagnoses were performed at the Cancer Pathology Laboratory, National Hospital Organization Kyushu Cancer Center and Department of Anatomic Pathology, Graduate School of Medical Sciences, Kyushu University Hospital, based on the 8th edition of the TNM classification. Pathological whole tumor size and pathological invasive size were evaluated by at least two pathologists. Patients were classified into NIA‐present and NIA‐absent groups according to the presence of NIAs, and the relationship between clinicopathological factors and prognosis was analyzed. Representative histological findings of the NIA‐present and NIA‐absent groups are shown in Figure 1.

### Statistical analysis

The Pearson's chi‐square test and Student's *t* test were used to analyze the difference in clinicopathological factors between NIA‐present and NIA‐absent groups. The DFS was defined as the period from surgery to the date of the last follow‐up, recurrence, or death, and OS was defined as the period from surgery to the date of the last follow‐up or death. The Kaplan–Meier method with the log‐rank test was used for plotting survival curves. Cox proportional hazards regression analysis was used to examine risk factors. In the multivariate analysis, only factors with *p* < 0.05 were extracted by the variable reduction method. JMP Pro 16.0 software (SAS Institute) was used for all statistical analyses.

### Propensity‐score matched analysis

A propensity‐score matched analysis was performed to reduce the bias between NIA‐present and NIA‐absent groups. The propensity scores comprised the following variables: age, sex, smoking history, surgical procedure, pathological invasive size, p‐T factor, p‐Stage, histological predominant subtype, ly, and v. A propensity score difference of 0.05 was adopted as the maximum caliper width for matching NIA‐present and NIA‐absent groups. Finally, 100 matched patients from each group were enrolled in the analysis.

## RESULTS

### Clinicopathological factors

A total of 402 patients with p‐Stage IA lung adenocarcinoma who underwent surgical resection were enrolled in this research. The clinicopathological factors of patients are shown in Supporting Information Table S1. The median age at surgery was 69 years (range 34–88 years). There were 173 men (43.0%) and 229 women (57.0%). In addition, 185 patients (46.0%) had a smoking history. The median radiological whole tumor size and solid component size were 19 mm (range 6–67 mm) and 12 mm (range 0–45 mm), respectively. The median C/T ratio was 0.67 (range 0.00–1.00), and GGO was present in 280 (69.7%) patients. In terms of surgical procedures, sublobar resection was performed on 91 patients (22.6%).

The median pathological whole tumor size and invasive area size were 17 mm (range 2–65 mm) and 13 mm (range 1–30 mm), respectively. On pathological diagnosis, 57 (14.2%), 89 (22.1%), 182 (45.3%), and 74 (18.4%) patients were diagnosed as p‐T1mi, p‐T1a, p‐T1b, and p‐T1c, respectively, and 146 (36.3%), 182 (45.3%), and 74 (18.4%) patients were diagnosed as p‐Stage IA1, IA2, and IA3, respectively. In terms of histological predominant subtypes, 86 (21.4%), 277 (68.9%), 16 (4.0%), and 23 (5.7%) patients had tumors with lepidic, acinar/papillary, solid/micropapillary, and other predominant adenocarcinomas, respectively. The positive rates of ly and v were 4 (0.1%) and 14 (3.5%), respectively. *EGFR* mutation was examined in 135 patients, and 85 (63.0%) patients had *EGFR* mutated lung adenocarcinoma. The median follow‐up period was 5.20 years (range 0.06–8.55 years).

### Clinicopathological factors according to the presence of NIAs

The numbers of patients in the NIA‐present and NIA‐absent groups were 231 (57.5%) and 171 (42.5%), respectively. The associations between the presence of NIAs and clinicopathological factors are demonstrated in Table 1. The Pearson's chi‐square test and Student's *t* test showed that NIA‐present was significantly associated with small radiological solid component size (*p* = 0.0042), smaller C/T ratio (*p* < 0.0001), GGO presence (*p* < 0.0001), larger pathological whole tumor size (*p* = 0.0055), smaller pathological invasive area size (*p* < 0.0001), earlier p‐T factor (*p* < 0.0001), earlier p‐Stage (*p* < 0.0001), LPA (*p* < 0.0001), v negativity (*p* = 0.0009), and *EGFR* mutation positivity (*p* = 0.0429).

**TABLE 1:** Clinicopathological factors according to the presence of noninvasive areas (NIAs)

Factors	NIA‐present (*N* = 231)	NIA‐absent (*N* = 171)	*p* value
Age, years	69 (38–87)	70 (34–88)	0.6088
Sex			
Male	100 (43.3%)	73 (42.7%)	0.9044
Female	131 (56.7%)	98 (57.3%)	
Smoking history			
Smoker	100 (43.3%)	85 (49.7%)	0.2018
Never smoker	131 (56.7%)	86 (50.3%)	
Radiological whole tumor size, mm	19 (7–67)	19 (6–52)	0.3530
Radiological solid component size, mm	11 (0–45)	15 (0–38)	0.0042
C/T ratio	0.58 (0.00–1.00)	0.53 (0.00–1.00)	<0.0001
Presence of GGO			
Present	181 (78.4%)	99 (57.9%)	<0.0001
Absent	50 (21.6%)	72 (42.1%)	
Surgical procedure			
Lobectomy	176 (76.2%)	135 (78.9%)	0.5137
Sublobar resection	55 (23.8%)	36 (21.1%)	
Pathological whole tumor size, mm	17 (5–65)	17 (2–30)	0.0055
Pathological invasive area size, mm	10 (1–30)	17 (2–30)	<0.0001
p‐T factor			
T1mi	57 (24.7%)	0 (0.0%)	<0.0001
T1a	60 (26.0%)	29 (16.9%)	
T1b	87 (37.7%)	95 (55.6%)	
T1c	27 (11.7%)	47 (27.5%)	
p‐Stage			
IA1	117 (50.6%)	29 (16.9%)	<0.0001
IA2	87 (37.7%)	95 (55.6%)	
IA3	27 (11.7%)	47 (27.5%)	
Histological predominant subtype			
Lepidic	86 (37.2%)	0 (0.0%)	<0.0001
Acinar/papillary	134 (58.0%)	143 (83.6%)	
Solid/micropapillary	5 (2.2%)	11 (6.4%)	
Others	6 (2.6%)	17 (9.9%)	
ly			
Negative	229 (99.1%)	169 (98.8%)	0.7616
Positive	2 (0.9%)	2 (1.2%)	
v			
Negative	229 (99.1%)	159 (93.0%)	0.0009
Positive	2 (0.9%)	12 (7.0%)	
*EGFR* mutation^a^			
Wild	21 (29.2%)	29 (46.0%)	0.0429
Mutation	51 (70.8%)	34 (54.0%)	

Abbreviations: C/T ratio, consolidation/tumor ratio; *EGFR*, epidermal growth factor receptor; GGO, ground glass opacity; ly, lymphatic invasion; p‐Stage, pathological stage; p‐T, pathological T; v, vascular invasion.

^a^ Available data were analyzed, excluding insufficient data.

### Prognosis analysis according to the presence of NIAs

**FIGURE 2:**
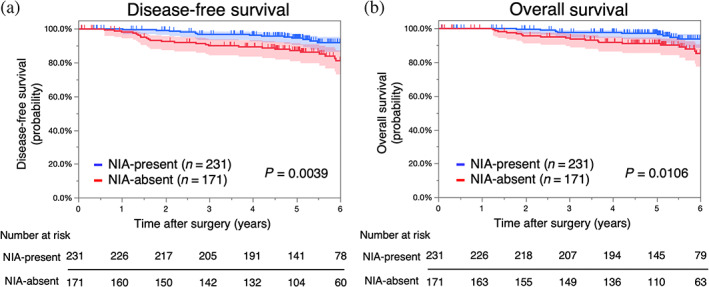
Survival curves of patients with lung adenocarcinoma according to the presence of NIAs before propensity‐score matching. (a) Disease‐free survival and (b) overall survival of NIA‐present and NIA‐absent groups. NIAs, noninvasive areas

Prognostic analyses according to the presence of NIAs were performed using the Kaplan–Meier method. The DFS and OS in the NIA‐present group were significantly better than those in the NIA‐absent group (5‐year DFS 94.6% vs. 87.2%, *p* = 0.0039, Figure 2a; 5‐year OS 97.2% vs. 91.1%, *p* = 0.0106, Figure 2b). However, the multivariable analysis showed that NIA‐present was not an independent prognostic factor for DFS and OS (Table 2).

**TABLE 2:** Univariable and multivariable analyses for the prognosis of pathological stage IA lung adenocarcinoma

Factors	Disease‐free survival						Overall survival					
	Univariable analysis			Multivariable analysis			Univariable analysis			Multivariable analysis		
	HR	95% CI	*p* value	HR	95% CI	*p* value	HR	95% CI	*p* value	HR	95% CI	*p* value
Age, years												
>70/<70	1.58	0.853–2.922	0.1461				2.38	1.142–4.970	0.0207			
Sex												
Male/female	2.10	1.132–3.892	0.0185				2.43	1.190–4.990	0.0151			
Smoking history												
Smoker/never smoker	2.53	1.329–4.817	0.0047	2.27	1.184–4.367	0.0136	2.85	1.345–6.043	0.0063	2.93	1.362–6.289	0.0059
Surgical procedure												
Lobectomy/sublobar resection	0.59	0.305–1.128	0.1099	0.33	0.169–0.664	0.0018	0.43	0.211–0.881	0.0212	0.30	0.141–0.625	0.0014
Presence of NIAs												
Present/absent	0.41	0.216–0.764	0.0052				0.40	0.192–0.827	0.0136			
p‐T factor												
T1b, T1c/T1mi, T1a	3.23	1.433–7.322	0.0047	2.91	1.194–7.112	0.0188	2.26	0.972–5.240	0.0584			
p‐Stage												
IA3/IA1, IA2	3.11	1.647–5.890	0.0005	2.43	1.231–4.795	0.0105	2.89	1.379–6.039	0.0049	3.29	1.527–7.102	0.0024
Histology												
non‐LPA/LPA	2.65	0.946–7.431	0.0637				1.88	0.658–5.355	0.2392			
ly												
Positive/negative	6.46	1.558–26.82	0.0102				3.14	0.428–23.08	0.2604			
v												
Positive/negative	5.05	1.978–12.90	0.0007				5.25	1.832–15.06	0.0020			

Abbreviations: 95% CI, 95% confidence interval; HR, hazard ratio; LPA, lepidic predominant adenocarcinoma; ly, lymphatic invasion; NIAs, noninvasive areas; non‐LPA, non‐lepidic predominant adenocarcinoma; p‐Stage, pathological stage; p‐T, pathological T; v, vascular invasion.

### Prognosis analysis according to the presence of NIAs after propensity‐score matched analysis

We then performed propensity‐score matched analysis to reduce the bias of clinicopathological factors between NIA‐present and NIA‐absent groups, and examined the prognostic impact of the presence of NIAs in more detail.

**FIGURE 3:**
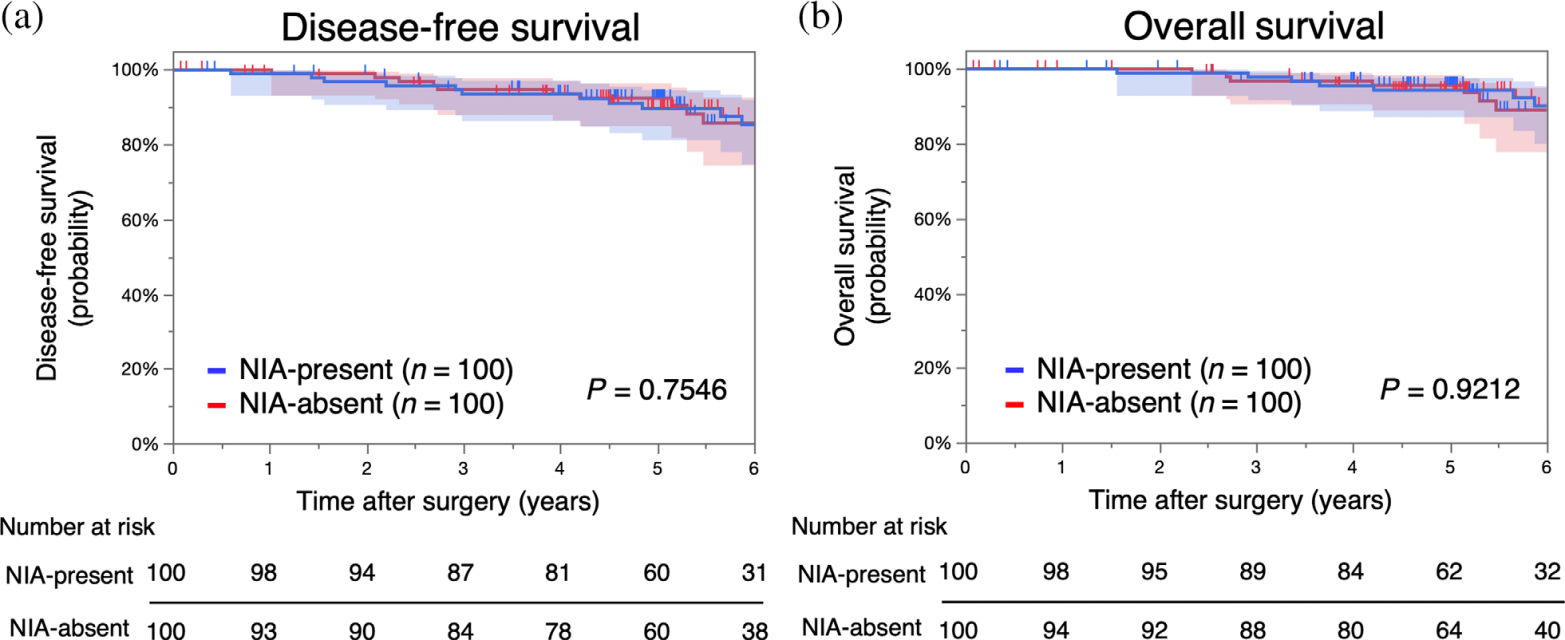
Survival curves of patients with lung adenocarcinoma according to the presence of NIAs after propensity‐score matching. (a) Disease‐free survival and (b) overall survival of NIA‐present and NIA‐absent groups. NIAs, noninvasive areas

After adjusting for the bias of clinicopathological factors by propensity‐score matched analysis, the Fisher's exact test and Student's *t* test demonstrated that most clinicopathological factors between the NIA‐present and NIA‐absent groups showed no significant difference (Table 3). Furthermore, Kaplan–Meier survival curves revealed that the prognostic differences in DFS and OS between the NIA‐present and NIA‐absent groups disappeared (5‐year DFS 92.4% vs 89.6%, *p* = 0.7546, Figure 3a; 5‐year OS 95.6% vs 94.3%, *p* = 0.9212, Figure 3b).

**TABLE 3:** Clinicopathological factors according to the presence of noninvasive areas (NIAs) after propensity‐score matching

Factors	NIA‐present (*N* = 121)	NIA‐absent (*N* = 121)	*p* value
Age, years	70 (46–85)	70 (40–88)	0.7958
Sex			
Male	44 (44.0%)	43 (43.0%)	0.8866
Female	56 (56.0%)	57 (57.0%)	
Smoking history			
Smoker	49 (49.0%)	46 (46.0%)	0.6710
Never smoker	51 (51.0%)	54 (54.0%)	
Radiological whole tumor size, mm	22 (9–51)	18 (6–37)	0.0006
Radiological solid component size, mm	15 (0–45)	14 (0–33)	0.3424
C/T ratio	0.67 (0.00–1.00)	0.82 (0.00–1.00)	0.2122
Presence of GGO			
Present	73 (73.0%)	54 (54.0%)	0.0053
Absent	27 (27.0%)	46 (46.0%)	
Surgical procedure			
Lobectomy	83 (83.0%)	81 (81.0%)	0.7128
Sublobar resection	17 (17.0%)	19 (19.0%)	
Pathological whole tumor size, mm	20 (9–45)	15 (2–29)	<0.0001
Pathological invasive area size, mm	15 (6–30)	15 (2–29)	0.6766
p‐T factor			
T1mi	0 (0.0%)	0 (0.0%)	0.9328
T1a	18 (18.0%)	20 (20.0%)	
T1b	60 (60.0%)	58 (58.0%)	
T1c	22 (22.0%)	22 (22.0%)	
p‐Stage			
IA1	18 (18.0%)	20 (20.0%)	0.9328
IA2	60 (60.0%)	58 (58.0%)	
IA3	22 (22.0%)	22 (22.0%)	
Histological predominant subtype			
Lepidic	0 (0.0%)	0 (0.0%)	0.7077
Acinar/papillary	94 (94.0%)	91 (91.0%)	
Solid/micropapillary	3 (3.0%)	5 (5.0%)	
Others	3 (3.0%)	4 (4.0%)	
ly			
Negative	98 (98.0%)	98 (98.0%)	1.0000
Positive	2 (2.0%)	2 (2.0%)	
v			
Negative	98 (98.0%)	99 (99.0%)	0.5607
Positive	2 (2.0%)	3 (3.0%)	
*EGFR* mutation^a^			
Wild	8 (24.2%)	16 (43.2%)	0.0946
Mutated	25 (75.8%)	21 (56.8%)	

Abbreviations: C/T ratio, consolidation/tumor ratio; *EGFR*, epidermal growth factor receptor; GGO, ground glass opacity; ly, lymphatic invasion; p‐Stage, pathological stage; p‐T, pathological T; v, vascular invasion.

^a^ Available data were analyzed, excluding insufficient data.

## DISCUSSION

**FIGURE 4:**
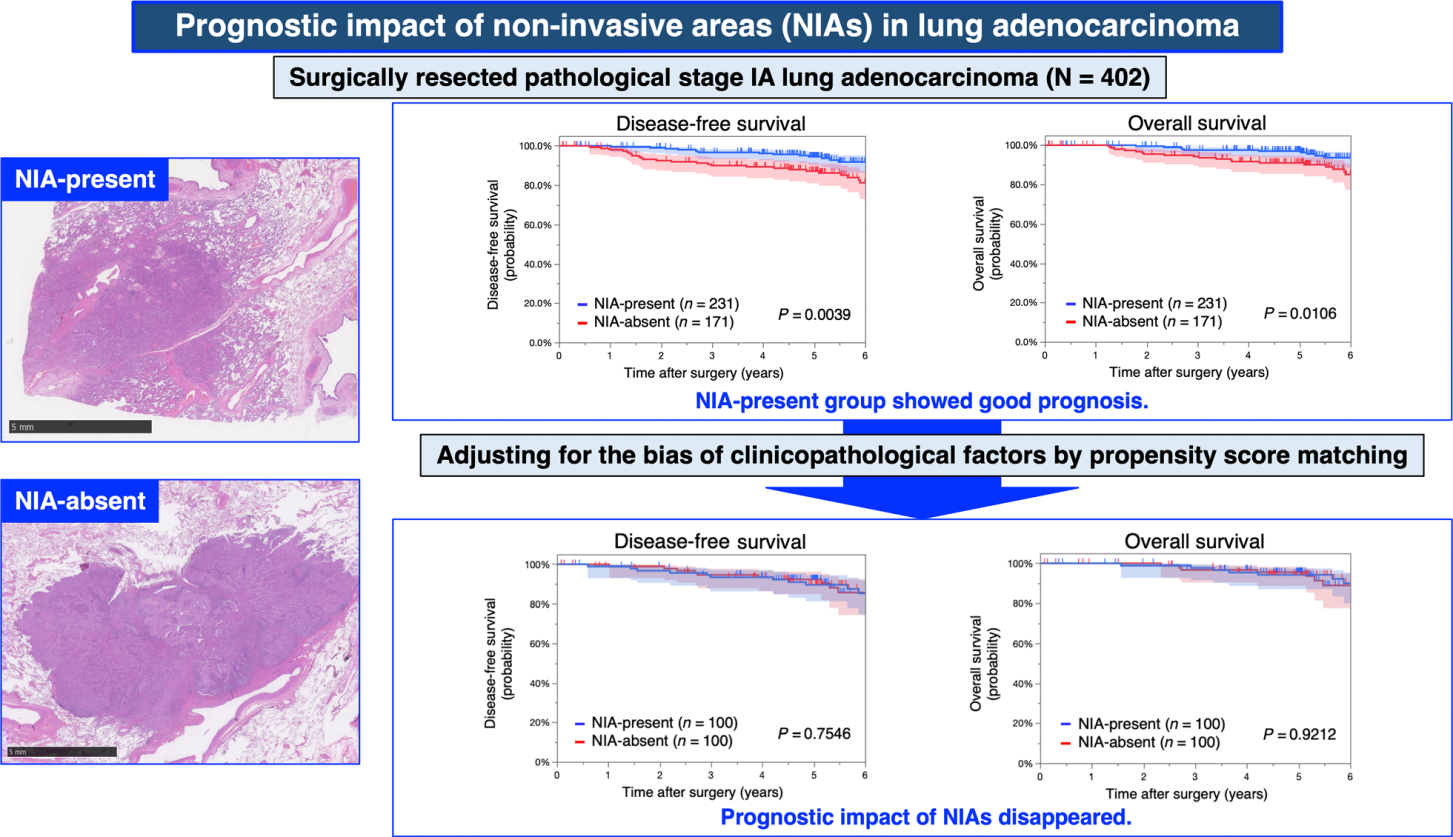
Summary of this study

In the current research, we described the prognostic impact of NIAs in surgically resected p‐Stage IA lung adenocarcinoma. The prognosis of the NIA‐present group was significantly better than that of the NIA‐absent group; however, after adjusting for the bias of clinicopathological factors by propensity‐score matched analysis, the prognostic impact of NIAs disappeared (Figure 4).

In terms of the association between the presence of NIAs and clinicopathological factors, NIA‐present was significantly associated with the presence of GGO, early stage, LPA, v negativity, and *EGFR* mutation positivity in our research. In particular, multivariable analysis showed that earlier p‐T factor, LPA, and *EGFR* mutation positivity were strongly associated with NIA‐present. These results were similar to the association between the presence of GGO and clinicopathological factors in previous reports that demonstrated the prognostic significance of GGO in NSCLC.^8^, ^9^, ^10^, ^11^, ^12^, ^13^, ^14^, ^15^ Therefore, our research appeared to be consistent with previous reports that the presence of radiological GGO reflected the presence of pathological NIAs.

Several reports have shown that the presence of radiological GGO is a favorable prognostic factor for NSCLC.^8^, ^9^, ^10^, ^11^, ^12^, ^13^, ^14^ In those reports, the GGO‐present group tended to have tumors with pathologically low‐grade malignancy, lepidic adenocarcinoma, and *EGFR* mutation,^8^, ^9^, ^10^, ^11^, ^12^, ^13^, ^14^ and the GGO‐absent group sometimes included histological types with poorer prognoses than adenocarcinoma, such as squamous cell carcinoma.^8^, ^9^ As a result, the presence of GGO has been reported as a favorable factor for NSCLC. Although many reports have analyzed the results without adjusting for background factors, some reports that adjusted for clinicopathological factors, such as histological features, using propensity‐score matching have suggested that the presence of GGO may not directly affect prognosis.^16^, ^17^ In addition, a previous study demonstrated that the expression of program death ligand‐1 protein on cancer cells was higher in GGO‐absent tumors than in GGO‐present tumors, and the tumor microenvironment may differ according to the presence of GGO.^18^ On the basis of these reports, we think that the presence of GGO may be useful in predicting histological features, but GGO itself cannot directly influence the prognosis of NSCLC. Moreover, the results of our present study showed that the presence of pathological NIAs had no prognostic impact after adjusting for clinicopathological factors by propensity‐score matched analysis. These results suggest that radiological GGO and pathological NIAs themselves might not directly impact the prognosis of lung adenocarcinoma.

As demonstrated in this study, NIA‐present lung adenocarcinomas often contain lepidic growth components. Regarding the prognostic impact of the presence of lepidic components in lung adenocarcinoma, some reports showed that their presence is a favorable prognostic factor,^19^, ^20^ whereas other reports demonstrated that lepidic components are not an independent prognostic factor in multivariable analysis and do not affect the prognosis of lung adenocarcinoma.^21^, ^22^ Our results showing that NIAs do not influence the prognosis of lung adenocarcinoma may support reports that there are minimal effects of lepidic components on the prognosis of lung adenocarcinoma.

Some previous reports suggested that the TNM classification at the clinical stage should be reconsidered according to the presence of GGO.^9^, ^10^, ^23^ However, considering that the prognostic impact of GGO and NIAs is strongly influenced by other histological features, and although the presence of radiological GGO is possibly useful to assess clinical stages where the histological features may not yet be fully known, the presence of pathological NIAs does not appear to be useful to assess p‐Stage where the histological features may already be known. The results of our study might support the usefulness of the 8th edition of the TNM classification, which excludes NIAs in the assessment of T and suggests that clinicians should be careful about adding GGO and NIAs to the TNM classification.

This study had several limitations. Because this research was retrospective with a limited number of patients, a large‐scale prospective study in the future is desired. In addition, the data on pathological invasive area size were assessed only from 2013, and the observation period of this study was relatively short. Moreover, the insufficient number of patients made it difficult to examine more detailed histological subtypes of lung adenocarcinoma in this study.

## CONCLUSION

Although the presence of NIAs was associated with a better prognosis of lung adenocarcinoma, the prognostic impact of NIAs disappeared after adjusting for the bias of clinicopathological factors by propensity‐score matched analysis. Our current study suggests that the prognostic impact of NIAs on lung adenocarcinoma is due to differences in clinicopathological factors.

## AUTHOR CONTRIBUTIONS

Conceptualization: F.K. and T.T. Data curation: F.K., T.T., T.O., K.T., and Y.O. Formal analysis: F.K., M.S., and T.T. Funding acquisition: F.K., T.T., T.O., and T.Y. Investigation: F.K., M.S., T.T., T.O., K.T., and Y.O. Methodology: F.K., M.S., and T.T. Project administration: F.K., T.T., T.O., Y.O., and T.Y. Resources: F.K., T.T., T.O., and T.Y. Software: F.K. and M.S. Supervision: M.S., T.T., Y.O., and T.Y. Validation: F.K., M.S., T.T., T.O., K.T., and Y.O. Visualization: F.K., M.S., and T.T. Roles/writing – original draft: F.K., M.S., and T.T. Writing – review & editing: F.K., M.S., T.T., T.O., K.T., Y.O., and T.Y.

## CONFLICT OF INTEREST STATEMENT

There is no conflict of interest to declare.

## Supporting information


**SUPPORTING INFORMATION TABLE S1.** Clinicopathological factors of patients with pathological stage IA lung adenocarcinoma

## Data Availability

The data underlying this article will be shared on reasonable request to the corresponding author.
