# Supplementary material for: Prognostic impact of noninvasive areas in resected pathological stage IA lung adenocarcinoma
Source: Thorac Cancer. 2023 Apr 27;14(18):1651–9. doi: 10.1111/1759-7714.14910 (PMC10290915; doi:10.1111/1759-7714.14910)
Supplement: Supplementary file 1 — SUPPORTING INFORMATION TABLE S1. Clinicopathological factors of patients with pathological stage IA lung adenocarcinoma [file TCA-14-1651-s001.docx]

**Supplementary Table 1. Clinicopathological factors of patients with pathological stage IA lung adenocarcinoma**

| Factors |  | Median (range) / Number (%) |
| --- | --- | --- |
| Age, years |  | 69 (34 - 88) |
| Sex | Male | 173 (43.0%) |
|  | Female | 229 (57.0%) |
| Smoking history | Smoker | 185 (46.0%) |
|  | Never smoker | 217 (54.0%) |
| Radiological whole tumor size, mm |  | 19 (6 - 67) |
| Radiological solid component size, mm |  | 12 (0 - 45) |
| C/T ratio |  | 0.67 (0.00 - 1.00) |
| Presence of GGO | Present | 280 (69.7%) |
|  | Absent | 122 (30.3%) |
| Surgical procedure | Lobectomy | 311 (77.4%) |
|  | Sublobar resection | 91 (22.6%) |
| Pathological whole tumor size, mm |  | 17 (2 - 65) |
| Pathological invasive area size, mm |  | 13 (1 - 30) |
| Presence of NIAs | Present | 231 (57.5%) |
|  | Absent | 171 (42.5%) |
| p-T factor | T1mi | 57 (14.2%) |
|  | T1a | 89 (22.1%) |
|  | T1b | 182 (45.3%) |
|  | T1c | 74 (18.4%) |
| p-Stage | IA1 | 146 (36.3%) |
|  | IA2 | 182 (45.3%) |
|  | IA3 | 74 (18.4%) |
| Histological predominant subtype | Lepidic | 86 (21.4%) |
|  | Acinar/Papillary | 277 (68.9%) |
|  | Solid/Micropapillary | 16 (4.0%) |
|  | Others | 23 (5.7%) |
| ly | Negative | 398 (99.0%) |
|  | Positive | 4 (0.1%) |
| v | Negative | 388 (96.5%) |
|  | Positive | 14 (3.5%) |
| *EGFR* mutation* | Wild | 50 (37.0%) |
|  | Mutation | 85 (63.0%) |

* Available data were analyzed, excluding insufficient data.

C/T ratio, consolidation/tumor ratio; GGO, ground glass opacity; NIAs, non-invasive areas; p-T, pathological T; p-Stage, pathological stage; ly, lymphatic invasion; v, vascular invasion; *EGFR,* epidermal growth factor receptor
